# Expression of Concern: MicroRNA-505-5p functions as a tumor suppressor by targeting Cyclin-Dependent Kinase 5 in cervical cancer

**DOI:** 10.1042/BSR-20191221_EOC

**Published:** 2020-07-02

**Keywords:** Cervical cancer, cell invasion, cell migration, cell proliferation, CDK5, miR-505-5p

The Editorial Office has been made aware of potential issues surrounding the scientific validity of this paper, hence has issued an expression of concern to notify readers whilst the Editorial Office investigates.

